# Combining Fourier Transform Infrared and Raman Spectroscopy to Characterize Kerogen Molecular Structures: Insights into Their Thermal Maturity

**DOI:** 10.3390/ijms26062696

**Published:** 2025-03-17

**Authors:** Dun Wu, Chenglong Wang, Wenxu Liang, Xia Gao

**Affiliations:** 1 Key Laboratory of Intelligent Underground Detection Technology, College of Civil Engineering, Anhui Jianzhu University, Hefei 230601, China; w2294080852@163.com; 2 School of Earth and Space Sciences, University of Science and Technology of China, Hefei 230026, China; 3 China Coal Research Institute, Beijing 100013, China; wenxu_liang@163.com; 4 School of Architecture & Urban Planning, Anhui Jianzhu University, Hefei 230601, China; xiagao@ahjzu.edu.cn

**Keywords:** kerogen, FTIR, Raman, molecular structure, thermal maturity

## Abstract

This study systematically analyzed the composition of organic functional groups and changes in the carbon structure of kerogen at different stages of thermal maturity using Fourier Transform Infrared (FTIR) spectroscopy and Laser Raman spectroscopy techniques. The research selected oil shale samples from the Late Carboniferous deep coal strata in the southern part of the Huainan coalfield. Kerogen was extracted through acid treatment, pyrite removal, and heavy liquid separation processes. Utilizing FTIR and Raman spectroscopy, the study delved into the quantitative and qualitative characteristics of functional groups such as hydroxyl, carboxyl, and methyl in the kerogen, as well as the variations in the ratio between aliphatic and aromatic carbon. The research found that as thermal maturity increased, aromatic structural parameters in the kerogen significantly rose, while aliphatic structural parameters exhibited a downward trend. Specifically, FTIR spectroscopy was used to identify the characteristic absorption wavenumber ranges of different functional groups and calculate key parameters such as the aromaticity of the kerogen and the ratio of aliphatic to aromatic functional groups using relevant formulas. Meanwhile, Raman spectroscopy analysis revealed changes in the orderliness of carbon atoms and the degree of graphitization in the kerogen as a function of thermal maturity, with the intensity ratio of the G band to the D_1_ band (A_D1_/A_G_) emerging as an important indicator for assessing thermal maturity. Additionally, this study further validated the correlation between thermal maturity and parameters such as reflectance (R_0_) and the H/C ratio by calculating the metamorphic temperature of the kerogen. Combining the results of FTIR and Raman spectroscopy analyses, this study unveiled a close relationship between the thermal maturity of kerogen and its organic functional group composition and carbon structure. As thermal maturity increased, the degree of aromatization in the kerogen rose, aliphatic chain lengths shortened, and the degree of graphitization improved. These findings not only enhance the understanding of the thermal evolution process of kerogen but also provide crucial scientific insights for oil and gas exploration and development.

## 1. Introduction

Kerogen refers to the large assemblage of insoluble organic molecules widely distributed across various sedimentary rock formations, representing one of the most abundant and ubiquitous forms of insoluble macromolecular organic matter on Earth [1,2]. As the original precursor of fossil fuels such as oil, natural gas, and oil shale [3], kerogen is not only the largest and economically significant organic carbon reservoir within underground hydrocarbon deposits but also a crucial element in maintaining Earth’s energy cycle and ecological balance [4]. Delving into the study of kerogen’s formation conditions, geological evolution, hydrocarbon-generating potential, and its relationship with reservoir and caprock formations holds significant strategic importance for oil and gas exploration and development. Specifically, kerogen research enables precise assessment of source rock maturity, which is a pivotal indicator for determining the quality of hydrocarbon resources and the difficulty of extraction; concurrently, it allows for accurate estimation of the total potential recoverable oil and gas resources, which is vital for formulating scientifically sound resource development strategies.

Research into kerogen encompasses a variety of advanced methodologies, including In Situ Combustion (ISC) [5,6], Fourier Transform Infrared Spectroscopy (FTIR) [7,8,9,10,11], X-Ray Diffraction (XRD) [12,13,14,15,16], Raman spectroscopy [17,18,19,20,21], Nuclear Magnetic Resonance (NMR) [12,22,23,24,25], and Scanning Electron Microscopy (SEM) [26,27]. These techniques provide robust support and a detailed perspective for in-depth exploration and structural analysis of kerogen. Zheng et al. [28] found that ISC technology can effectively enhance oil production rates by increasing gas injection rates and oxygen content. Wu et al. [29] using FTIR and XRD, demonstrated that magma intrusion alters kerogen, leading to coal metamorphism that significantly changes the chemical structure of coal. Kelemen [30] explored the potential of Raman spectroscopy in providing information about the maturity of kerogen and coal during catalytic stages. Mao et al. [31] utilized advanced quantitative solid-state NMR technology to study structural changes in a series of Type II kerogen samples from the New Albany Shale, confirming that aromaticity is an excellent NMR structural parameter for assessing thermal maturity. Liu et al. [32], based on SEM observations of highly reactive shale organics, were able to determine the thermal maturity and composition of the original organics.

However, each of these methods has its own limitations. ISC technology is costly and complex to implement; XRD technology has low sensitivity when detecting light elements and is susceptible to interference and overlapping peaks; NMR technology requires a closed environment and is prone to noise interference; and SEM technology may suffer from electrostatic field interference due to charged samples, affecting image quality. In contrast, FTIR technology boasts excellent reproducibility, fast scanning speed, high resolution, high sensitivity, and a broad spectral range for research. Raman spectroscopy is renowned for its non-destructiveness, simplicity in sample preparation, specificity, and complementarity. Consequently, FTIR and Raman spectroscopy are widely used to characterize the organic functional group composition and carbon structure of kerogen. Delano et al. [33] successfully determined the vitrinite reflectance equivalent (VREO) maturity value of organics in petroleum exploration using Raman spectroscopy, providing new insights into this field. He et al. [34] combined FTIR and Raman spectroscopy to thoroughly evaluate the distribution of functional groups and the evolution of chemical structures in different grades of coal samples during coalification.

The aim of this study is to deeply explore the impact of oil shale thermal maturity on the organic functional group composition and carbon structure of kerogen, systematically analyze the chemical transformation mechanisms of kerogen during the thermal maturity process [35], and assess the indicative significance of these changes for the geological characteristics of the host rocks. The research focuses on the evolution characteristics of organic functional groups in kerogen samples as they undergo changes in thermal maturity stages, encompassing quantitative and qualitative analyses of functional groups such as hydroxyl, carboxyl, and methyl groups, as well as carbon structure analysis of aliphatic and aromatic carbon ratio changes, mapping out the molecular structural transformation pathways during the thermal evolution of kerogen [36]. By investigating the correlation between kerogen thermal maturity and its organic functional group composition and carbon structure, we can assess the thermal maturity state of kerogen, thereby effectively improving the prediction accuracy of its hydrocarbon generation potential and hydrocarbon generation time windows.

## 2. Results

### 2.1. Basic Properties and Characteristics of Kerogen Samples

Based on the data presented in Table 1, the maximum kerogen content is found in sample M-5, weighing 32 g, while the minimum is in sample M-8, weighing 11 g. The average content for all samples is 17.5 g. The highest R_O_ value belongs to sample M-1, at 1.15%, with the lowest value recorded in sample M-2, at 0.99%. The average R_O_ value for all samples is 1.09%. Except for sample M-8, which has a significantly elevated O/C content, the O/C contents of the other samples are all around 0.1. The maximum H/C ratio is observed in sample M-2, at 1.10, with the lowest values seen in samples M-3 and M-5, both at 0.73. The average H/C ratio for all samples is approximately 0.84. The chemical composition of kerogen primarily consists of elements such as carbon, hydrogen, oxygen, nitrogen, and sulfur, with carbon and hydrogen being the most dominant. This high content of carbon and hydrogen gives kerogen its unique molecular structure and rich chemical properties. Kerogen’s molecular structure is incredibly complex, far from that of a single compound; it is a highly ordered mixture composed of various functional groups [35]. The H/C ratio of kerogen is one of the critical parameters for assessing its hydrocarbon-generating potential. A higher H/C ratio typically indicates that the kerogen contains more aliphatic structural units (like alkanes and cycloalkanes) or aromatic compounds rich in hydrogen substituents. These compounds are more easily converted into hydrocarbon gases and liquids during pyrolysis or hydrocracking. Research by Wang [37] shows that a carbon-to-hydrogen atomic ratio greater than 0.8 can be defined as hydrogen-rich. When the H/C ratio of kerogen exceeds 0.8, meaning its molecular structure contains more hydrogen elements, such kerogen is referred to as hydrogen-rich kerogen.

### 2.2. Infrared Spectral Characteristics of Kerogen

**Figure 1:**
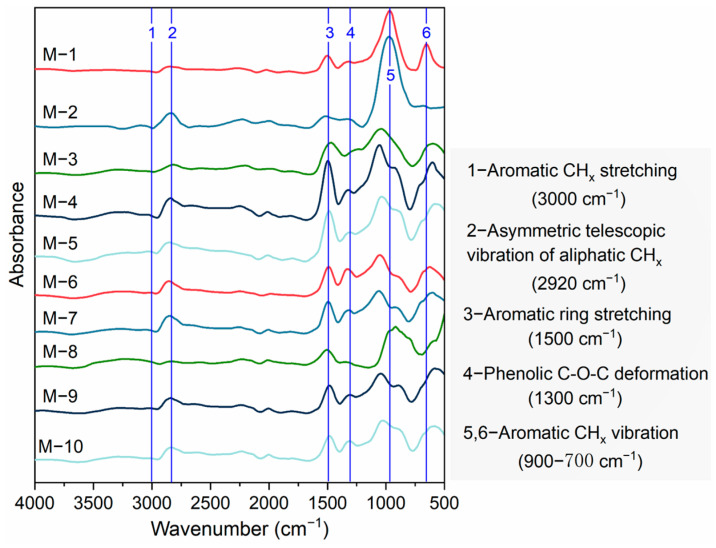
FTIR spectrum of kerogen sample (after smooth normalization processing).

Figure 1 displays the FTIR spectral curves of ten oil shale samples. Near 3000 cm^−1^, samples M-2, M-4, M-5, and M-8 exhibit insignificant absorption peaks compared to other oil shale samples, with M-8 peak shifted to the left, potentially related to the presence of vitrinite components in M-8. Around 2920 cm^−1^, samples M-1, M-5, M-6, M-9, and M-10 exhibit prominent absorption peaks compared to other oil shale samples, indicating that these samples possess longer aliphatic CH_x_ structures and undergo asymmetric stretching vibrations [38]. In the vicinity of 1500 cm^−1^, samples M-1 and M-6 show distinct absorption peaks compared to other oil shale samples, possibly due to their high content of various rock components. In the spectral region near 1300 cm^−1^, samples M-6 and M-9 exhibit significant absorption peaks, which may be associated with the higher organic matter content in these two samples. Near the range of 900 to 700 cm^−1^, samples M-1, M-2, and M-3 present clear absorption peaks compared to other oil shale samples, suggesting that these samples have longer C-H structures and undergo bending vibrations.

**Figure 2:**
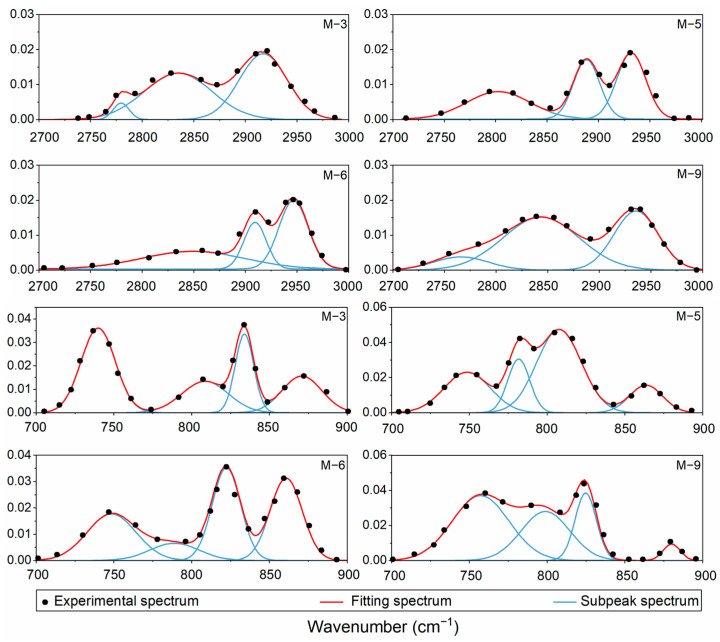
FTIR fitting analysis at different wavelengths.

Gauss function deconvolution fitting was performed on the two wavelength bands of 700–900 cm^−1^ and 2700–3000 cm^−1^ using Origin 2018 software, with the aim of extracting key spectral parameters. Prior to the Gauss function fitting, the raw spectral data underwent preprocessing, which included smoothing for noise reduction and baseline correction, to mitigate any adverse effects that instrumental noise and background interference might have on the fitting results. Subsequently, a multi-iterative fitting approach was adopted, involving gradual adjustments to parameters such as the number, position, and FWHM of the Gauss functions, until the fitting residual reached a minimum. This ensured that each fitted peak accurately reflected the vibrational characteristics of different chemical components in the sample. Finally, the spectral parameters obtained from the fitting (e.g., position, area, FWHM, etc.) will be used to further analyze the chemical composition and structural characteristics of the kerogen samples. Based on this, M-3, M-5, M-6, and M-9 were selected for deconvolution analysis of infrared spectra (Figure 2). According to Equations (1)–(6), the infrared spectral parameters of 10 kerogen samples are presented in Table 2.

#### 2.2.1. Evolution of Aromatic Compounds

**Figure 3:**
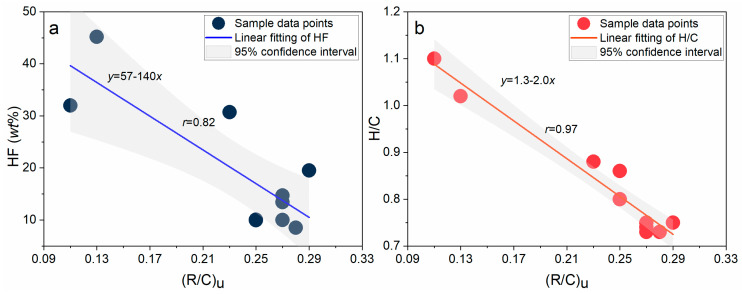
(**a**) Correlation between aromatic structural parameters and HF. (**b**) Correlation between aromatic structural parameters and H/C.

The characteristic absorption wavenumber ranges of aromatic compounds in infrared spectroscopy include 3100–3000 cm^−1^, 1600–1450 cm^−1^, and 900–700 cm^−1^. Specifically, the 3100–3000 cm^−1^ range corresponds to the stretching vibration of aromatic C-H bonds, the 1600–1450 cm^−1^ range involves the stretching vibration of C=C bonds and ring deformation vibrations, and the 900–700 cm^−1^ range represents the out-of-plane bending vibrations of aromatic rings [39]. The aromatic structural parameter has a maximum value of 0.29 and a minimum value of 0.11, with an average value of approximately 0.23 among the 10 samples. To investigate the correlation of the aromatic structural parameters among these 10 samples, correlation analyses were conducted between the aromatic structural parameter and both HF and H/C (Figure 3). It is evident that the aromatic structural parameter exhibits high correlations with both HF and H/C, with the correlation with H/C reaching as high as 0.97.

#### 2.2.2. Evolution of Aliphatic

**Figure 4:**
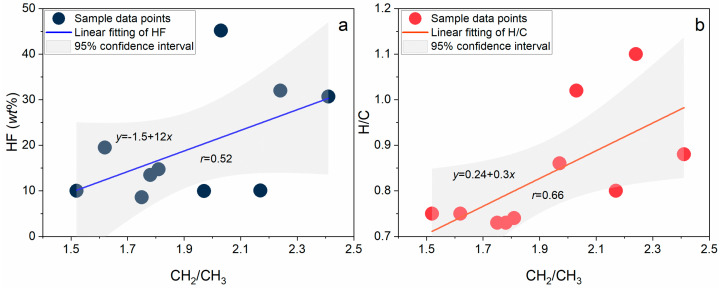
(**a**) Correlation between CH_2_/CH_3_ and HF. (**b**) Correlation between CH_2_/CH_3_ and H/C.

Based on the detailed CH_2_/CH_3_ ratio data listed in Table 2, it can be observed that M-10 has the highest ratio, reaching 2.41, while M-4 has the lowest ratio, at 1.52. The average CH_2_/CH_3_ ratio among the 10 samples is 1.93, with a standard deviation of approximately 0.33, indicating a certain degree of concentration in the data distribution. Specifically, M-6 has a CH_2_/CH_3_ ratio of 1.97, significantly higher than those of M-3 (1.75), M-5 (1.78), and M-9 (1.81). This difference is statistically significant as verified by the *t*-test (*p* < 0.05). This suggests that the aliphatic chain length of M-6 is significantly greater than that of M-3, M-5, and M-9, and the difference in aliphatic chain length can be quantified by calculating the difference in CH_2_/CH_3_ ratios among the samples. The difference between the samples is mainly reflected in the increase of CH_2_ groups, which is primarily due to the shortening of alkyl chains and the conversion of alkylmethylene structures to aromatic rings or branched aliphatic structures [29]. As shown in Figure 4, the CH_2_/CH_3_ ratio exhibits a positive correlation with both the hydrogen index (HF) and the hydrogen-to-carbon atomic ratio (H/C) to some extent. The correlation coefficient between CH_2_/CH_3_ and HF is 0.52, and between CH_2_/CH_3_ and H/C is 0.66. These correlations are statistically significant as verified by the Pearson correlation coefficient test (*p* < 0.05). Additionally, by comparing the correlation strengths in Figure 4a,b, it can be found that the correlation between CH_2_/CH_3_ and H/C is higher than that between CH_2_/CH_3_ and HF, indicating that changes in the CH_2_/CH_3_ ratio are more closely related to H/C, potentially associated with factors such as the type of organic matter, maturity, or thermal evolution degree of the samples. Overall, the content of methylene groups increases with the increase in H/C, which may be due to an enhanced condensation level of aromatic rings, leading to a higher degree of aromatization and a reduction in aromatic side chains and aliphatic structures.

#### 2.2.3. Evolution of Oxygen-Containing Structures

The spectral region between 1700 and 1000 cm^−1^ indicates the presence of oxygen-containing functional groups, and the functional groups vary among different grades of kerogen. Compared to the oxygen-containing functional group at the 1680 cm^−1^ wavelength band in the M-2 sample, M-1, M-3, M-4, M-6, M-7, and M-10 exhibit shifts toward lower-wavelength bands, while M-5, M-8, and M-9 shift toward higher bands, suggesting the conversion of aliphatic carbonyls to aromatic carbonyls [40]. M-3, M-4, and M-8 show significant peaks at the 1240 cm^−1^ wavelength band corresponding to the C-O-C structure, while other kerogen samples also exhibit shifts but lack the carbonyl stability observed in M-5, M-8, and M-9 kerogen samples [40].

### 2.3. Raman Spectral Characteristics of Kerogen Samples

**Figure 5:**
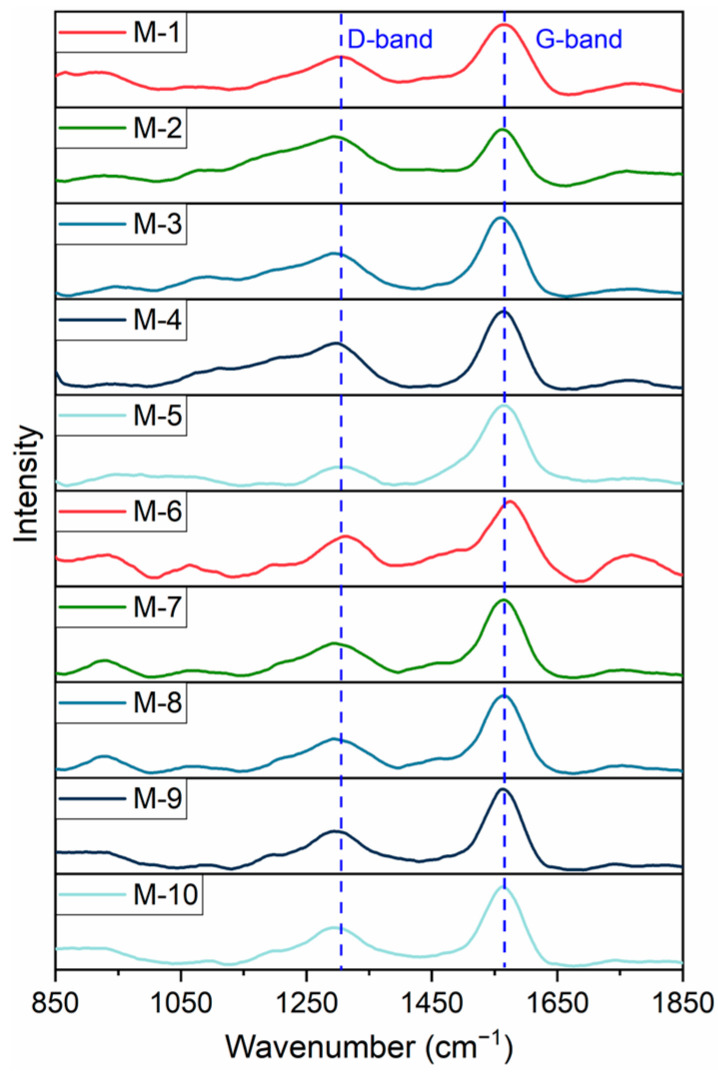
Raman spectrum of kerogen samples (after smooth normalization processing).

Figure 5 displays the original Raman spectra of ten kerogen samples. The figure reveals two distinct peaks for all ten samples, located approximately at 1300 cm^−1^ and 1590 cm^−1^, corresponding to the D_1_ and G bands, respectively. An increase in the H/C ratio of kerogen, which indicates a relative increase in hydrogen content, may lead to an increase in disorder or defects in the kerogen structure. In Raman spectroscopy, this may manifest as an increase in the intensity of the D band, resulting in a decrease in the graphitization structure or orderliness of the kerogen, and potentially weakening the intensity of the G band [39]. Since M-2, M-6, M-8, and M-10 are hydrogen-rich, with reduced graphitization or increased disorderliness, it is evident that their G band intensities are weakened. Conversely, M-3, M-5, and M-9 have relatively lower H/C contents, all below 0.75, indicating reduced graphitization or increased orderliness, which results in an enhancement of their G band intensities.

**Figure 6:**
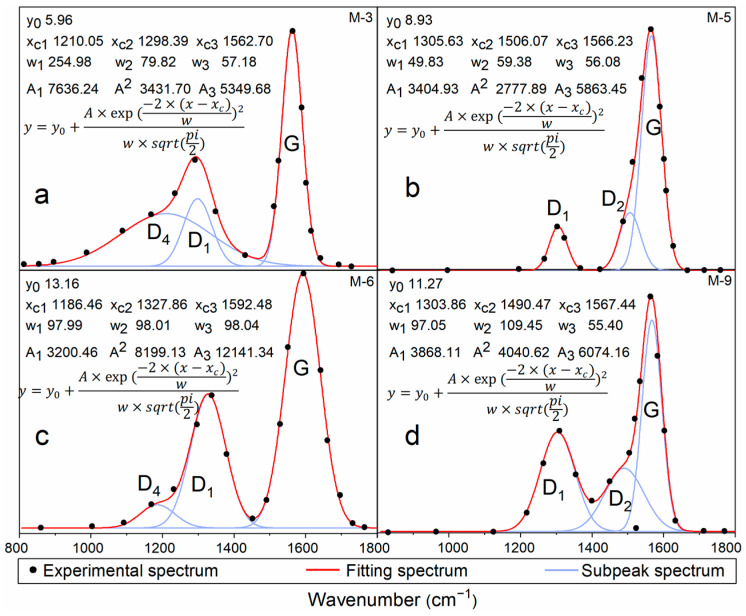
Gaussian fitting diagram of Raman spectra for four representative kerogen samples. (**a**–**d**) present the Gaussian function fitting results of the Raman spectra for four samples: M-3, M-5, M-6, and M-9, respectively. It can be observed that the fitting results for M-3 and M-6 are similar, both exhibiting a D_4_ peak; whereas samples M-5 and M-9 exhibit a D_2_ peak. In this figure, y_0_: the basic offset or baseline of the function; A: representing the amplitude, which is the difference between the peak value of the function and the baseline y_0_; w: the width parameter; sqrt(pi/2): a constant factor; x_c_: the center position, indicating the x-value where the peak of the function occurs.

To conduct an in-depth study of the Raman spectroscopic characteristics of kerogen samples, we selected four representative samples, namely M-3, M-5, M-6, and M-9, and performed a fitting analysis within the wavelength range of 800–1800 cm^−1^. The results are presented in Figure 6. Based on the fitted outcomes, we compiled the data in Table 3.

As can be seen from Table 2, the peak positions of the four kerogen samples (such as G and D_1_) are relatively close, mainly located near 1580 cm^−1^ and 1330 cm^−1^. This indicates that the selected kerogen samples share similar ordered structural characteristics. The peak position difference (θ) between the G peak and the D_1_ peak ranges from 260.39 cm^−1^ to 264.74 cm^−1^. Among the four kerogen samples, M-6 has the largest peak position difference, suggesting that its structural order is relatively low compared to the other samples, which may imply a lower level of maturity. The ratio of A_D1_/A_G_ ranges from 0.58 to 0.68, with M-6 having a significantly higher ratio, further confirming its relatively low thermal maturity. Additionally, M-3 has an A_D4_/A_G_ value of 1.43, M-5 has an A_D2_/A_G_ value of 0.47, M-6 has an A_D4_/A_G_ value of 0.26, and M-9 has an A_D2_/A_G_ value of 0.66. Combining this with Figure 6, we can infer that the differences among these four samples stem from their varying degrees of thermal maturity, leading to different contents and intensities of functional groups and thus fitting different peaks.

**Figure 7:**
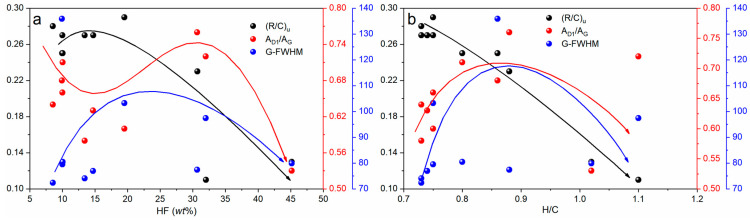
(**a**) Correlation analysis of HF with (R/C)_u_, A_D1_/A_G_ (R/C)_u_ and G-FWHM. (**b**) Correlation analysis of H/C with (R/C)_u_, A_D1_/A_G_ (R/C)_u_ and G-FWHM.

The specific property or compositional variations of HF exhibit a notable synchronous trend with parameters such as the position, intensity, and morphology of characteristic absorption peaks of aromatic compounds observed in infrared spectroscopy (e.g., (R/C)_u_). There is also a close correlation between the absorption intensity in infrared spectroscopy and the H/C value of kerogen. Numerous scholars [33,41,42,43,44] have reported that the ratio of the peak area or peak intensity between the G band and D band can serve as a key indicator for assessing kerogen maturity. We previously conducted research on the influence of the thermal behavior and Raman spectroscopic characteristics of hydrogen-rich coal on the process of thermal maturation, revealing that the FWHM can serve as an indicator for evaluating the degree of thermal evolution in perhydrous coal [39]. Zhou et al. [45] further pointed out that the positions of the D band and G band can also effectively reflect the metamorphic grade of coal. Additionally, research by Hara et al. [46] revealed a significant correlation between spectral band ratios calculated based on Raman spectroscopy and the metamorphic grade of different types of flint samples containing a certain amount of randomly distributed carbonaceous matter (CM). Hence, by observing changes in the width of the G peak in Raman spectroscopy, one can indirectly infer trends in the H/C ratio. This trend is closely related to the vibrational mode information of carbon nanomaterials or organic molecules revealed by Raman spectroscopy. Notably, HF is extremely sensitive to interlayer interactions within CM. When the H/C ratio exceeds 0.8, forming so-called “hydrogen-rich” kerogen, it leads to a decrease in graphitization degree, accompanied by a weakening of the G peak intensity or a shift towards lower frequencies. To delve deeper into the correlation between infrared and Raman spectroscopic parameters of kerogen from different samples, regression analysis was conducted (Figure 7), encompassing aromatic and aliphatic functional group parameters in infrared spectroscopy, the A_D1_/A_G_ ratio, the full width at half maximum (G-FWHM) of the G peak, as well as variables such as HF treatment and the H/C ratio. As shown in Figure 7a, with the increase of HF, (R/C)_u_ exhibits a trend of initially increasing and then gradually decreasing; the trend of A_D1_/A_G_ is to first decrease, then increase, and finally decrease again; whereas the trend of G-FWHM is to first increase and then gradually decrease. Figure 7b further reveals a negative correlation between (R/C)_u_ and H/C. As H/C increases, A_D1_/A_G_ also demonstrates a trend of first increasing and then gradually decreasing, with G-FWHM exhibiting the same trend as A_D1_/A_G_.

## 3. Discussion

**Figure 8:**
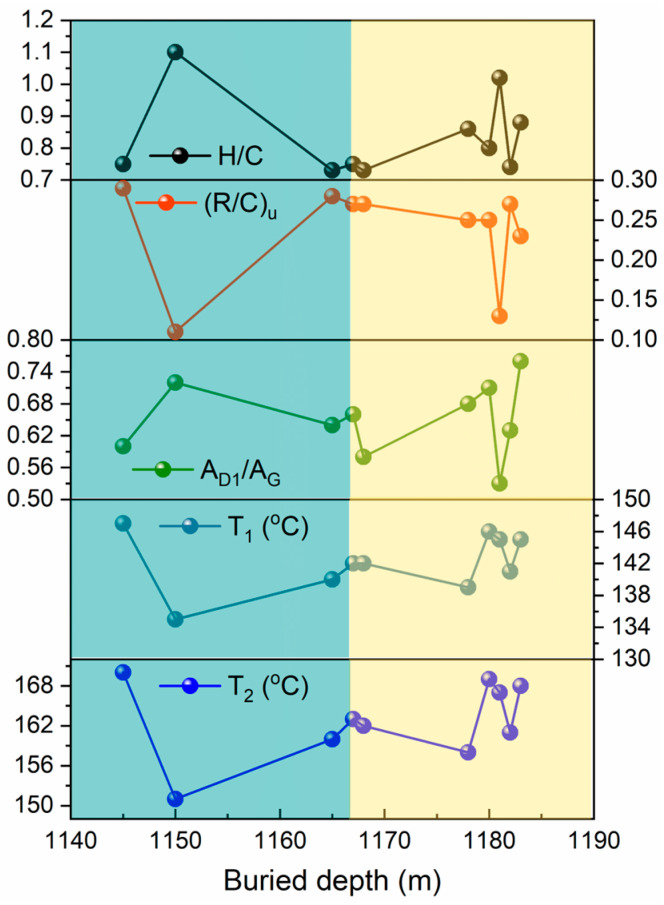
Correlation analysis of metamorphic temperature (T_1_ and T_2_), H/C, (R/C)u, and A_D1_/I_G_.

There exists a tight positive correlation between the metamorphic temperature of kerogen and its thermal maturity, where the level of the metamorphic temperature directly determines the degree of thermal maturity. This is because high temperature is one of the core factors triggering the metamorphism of kerogen. As the temperature gradually rises, the organic matter within the kerogen undergoes a series of complex chemical reactions, such as pyrolysis and condensation, which not only alter the chemical composition of the kerogen but also affect its physical properties, thereby enhancing its thermal maturity. Lundsdorf et al. [43] investigated the temperature range of metamorphic sedimentary rocks with carbonaceous material (CM) as the carrier, which is approximately between 160 °C and 600 °C. They proposed two temperature calculation formulas based on excitation wavelengths of 488 nm and 532 nm, aiming to estimate metamorphic temperatures using Raman spectroscopic data of carbonaceous materials. In contrast, the sample types studied by Beyssac et al. [47] mainly consisted of metamorphic slates and calcareous metamorphic sediments. The formulas they proposed, which are related to Raman spectroscopic parameters, are applicable to a temperature range of 330 °C to 650 °C. Geological thermometers offer new tools for studying regional metamorphism and have broad application prospects. Their research on temperature calculation formulas demonstrates that Raman spectroscopic characteristics exhibit good results in the high-temperature range. Based on the classification criteria proposed by Kouketsu et al. [41], the organic matter in the Taiyuan Formation exhibits an extremely low maturity level, being in the initial stage of amorphous carbon or transitioning towards graphitization, with the corresponding metamorphic temperature limited to below 300 °C. Therefore, for such low-maturity organic matter samples, it is advisable to select and use empirical formulas for calculating geothermal temperatures based on Raman vitrinite reflectance. Based on the research results of Barker and Pawlewicz [48], under different metamorphic conditions, the metamorphic temperatures of coal can be calculated using the following formulas: for burial metamorphism, T1 = [Ln(R_o_) + 1.68]/0.0124; for hydrothermal metamorphism, T2 = [Ln(R_o_) + 1.19]/0.00782. Using the data from Table 1, we further derived the metamorphic temperatures for the 10 samples (Table 4). In this formula, a clear positive correlation is observed between the reflectivity (R_o_) and the metamorphic temperature of kerogen. Consequently, Ro has become an important and crucial indicator reflecting the thermal maturity of kerogen samples. As the heating intensity of kerogen increases, significant changes occur in its internal organic structure, which are directly reflected in the values of Ro. Higher Ro values indicate a higher degree of thermal maturity for the kerogen, also confirming that M-5 and M-9 have higher thermal maturity than M-6. The correlation analysis of the degradation temperatures (T_1_ and T_2_), H/C, (R/C)_u_, and A_D1_/A_G_ for the 10 samples is shown in Figure 8.

The elemental composition of kerogen, including the content of elements such as carbon, hydrogen, oxygen, nitrogen, and sulfur, is closely related to its thermal maturity. As thermal maturity increases, the hydrogen content in kerogen gradually decreases, while the carbon content relatively increases. Specifically, the hydrogen-to-carbon atomic ratio (H/C) of M-6 is 0.86, higher than that of M-5 at 0.73 and M-9 at 0.74. Therefore, M-6 is classified as hydrogen-rich kerogen with a relatively high hydrogen content in its organic matter. This generally indicates a lower degree of metamorphism for M-6, reflecting a correspondingly lower thermal maturity. In contrast, M-5 and M-9 have higher reflectivity values (R_0_) of 1.08% and 1.07%, respectively, indicating they have undergone higher metamorphic temperatures and thus possess higher thermal maturity. Meanwhile, M-3 and M-6 have reflectivity values (R_0_) of 1.06% and 1.05%, respectively, suggesting lower metamorphic temperatures and slightly lower thermal maturity. Additionally, M-6 has the highest A_D1_/A_G_ value of 0.68, indicating a more disordered structure with more defects, further pointing to a relatively lower thermal maturity. Conversely, M-3, M-5, and M-9 have lower A_D1_/A_G_ values, suggesting a higher degree of graphitization and thus higher thermal maturity. Figure 8 demonstrates the correlations between burial depth and metamorphic temperatures (T_1_ and T_2_), H/C ratio, (R/C)_u_, and A_D1_/A_G_. As burial depth increases, the metamorphic temperatures (T_1_ and T_2_) exhibit a trend of decrease–increase–decrease–increase–decrease–increase again. Simultaneously, the trend of (R/C)_u_ is completely opposite to that of H/C; however, in most samples, the trends of (R/C)_u_ and H/C are the same, with opposing trends only observed in sections M-6 to M-9. On the other hand, as metamorphic temperatures rise, the thermal maturity of kerogen also increases, leading to enhanced ordering of carbon atoms within the kerogen. This is typically manifested as an increase in the intensity of the G peak and a decrease in the A_D1_/A_G_ value. In summary, M-5 and M-9 exhibit higher thermal maturity, while M-3 and M-6 show lower thermal maturity. It is noteworthy, however, that although the differences in depth and metamorphic temperatures shown in Figure 8 may not be numerically significant, these slight variations can, to some extent, reveal differences in spectral characteristics, elemental properties, and organic matter content among the kerogen samples. This study also indicates that spectral measurement techniques can be utilized to characterize the thermal maturity of kerogen samples.

## 4. Sample Collection and Experimental Analysis

### 4.1. Geological Background

**Figure 9:**
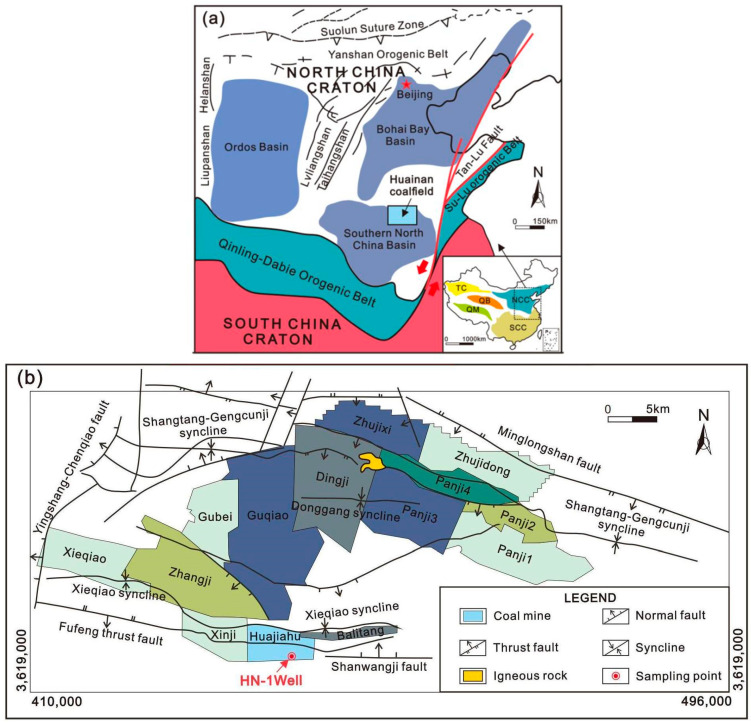
(**a**) Distribution map of major basins in North China [49]; (**b**) geological structure map of Panxie mining area, Huainan coalfield.

The Huainan coalfield is located on the southern edge of the North China Craton Basin, with an overall elongated oval shape, approximately 180 km in length and between 20 and 30 km in width, covering a geographical area of roughly 3654 km^2^ (Figure 9). The coal-bearing strata in the Huainan coalfield are mainly Carboniferous–Permian formations of the North China type, with the lithostratigraphic units, from bottom to top, being the Benxi Formation, Taiyuan Formation, Shanxi Formation, Lower Shihezi Formation, and Upper Shihezi Formation. Most of the coal-bearing strata in the Huainan coalfield are covered by Mesozoic and Cenozoic formations. The thickness of the overlying strata gradually increases from northeast to southwest, typically ranging from 100 to 600 m based on drilling and mine development data. However, in the western part of the Huainan coalfield, drilling results indicate that the thickness of the overlying strata exceeds 3000 m. Previous geological work has primarily focused on geological exploration and the investigation, evaluation, and exploration of coalbed methane resources in the Huainan coalfield, with the main target formations being the Upper Shihezi Formation, Lower Shihezi Formation, and Shanxi Formation of the Permian. Due to the Taiyuan Formation generally being buried deeply and consisting mostly of thin coal seams, it is usually not considered a primary horizon for coal resource exploration, and drilling efforts targeting the Taiyuan Formation are relatively limited. The HN-1 well is the first parametric well for evaluating the unconventional natural gas development potential of the coal-bearing strata of the Late Carboniferous Taiyuan Formation in the Huainan coalfield. Spectroscopic analysis of the oil shale samples obtained from drilling cores of this well can provide further insights into the maturity and hydrocarbon generation potential of the oil shale.

### 4.2. Sample Collection

**Figure 10:**
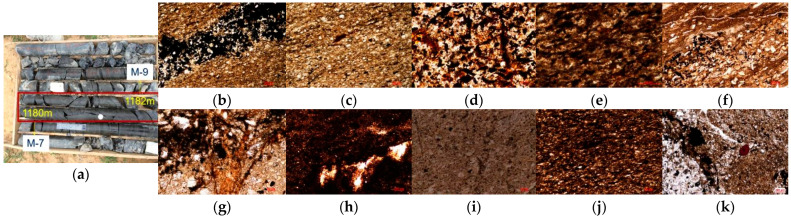
(**a**) Core interval at 1180–1182 m of the HN-1 well; (**b**) M-1, dark mudstone with silty composition, containing numerous vitrinite macerals (transmitted light, ×100); (**c**) M-2, dark mudstone with silty composition, and abundant vitrinite macerals visible (transmitted light, ×100); (**d**) M-3, abundant vitrinite microcomponents observed (transmitted light, ×50); (**e**) M-4, silty mudstone with extensive vitrinite development, and abundant vitrinite development (transmitted light, ×50); (**f**) M-5, silty mudstone intercalated with argillaceous siltstone, and pyrite development visible (transmitted light, ×200); (**g**) M-6, silty mudstone often containing many vitrinite macerals (transmitted light, ×50); (**h**) M-7, rich in organic matter, primarily vitrinite (transmitted light, ×200); (**i**) M-8, containing vitrinite macerals (transmitted light, ×50); (**j**) M-9, higher content of dark organic components, with silt included (transmitted light, ×50); (**k**) M-10, well-developed vitrinite, containing resinite (transmitted light, ×200).

The sampling site is located at the HN-1 well in the Huajiahu coal mine, the Panxie mining area of the Huainan coalfield (Figure 9b), which is the only parametric well in the Huainan coalfield where the entire stratigraphic core of the Taiyuan Formation has been drilled. Using wireline coring technology, various types of source rock samples, including limestone, oil shale, and coal, were collected from the coal-bearing strata of the Taiyuan Formation at depths ranging from approximately 1130 m to 1240 m. Among them, the stratigraphic interval from 1145 m to 1185 m is the main enrichment area for oil shale samples. The samples are numbered in order of depth from shallow to deep as M-1 (1145 m), M-2 (1150 m), M-3 (1165 m), M-4 (1167 m), M-5 (1168 m), M-6 (1178 m), M-7 (1180 m), M-8 (1181 m), M-9 (1182 m), and M-10 (1183 m). Figure 10a shows the coring interval from 1180 to 1182 m, where it can be clearly observed that the surfaces of the M-7 and M-9 oil shale samples are smooth, dense, and black, with oil-like substances flowing out, and a distinct petroleum-like odor can be detected. Moreover, to gain a deeper understanding of the petrological characteristics of the 10 collected oil shale samples, morphological observations were conducted using a polarized light microscope (Figure 10b–k).

### 4.3. Kerogen Extraction

In this study, the procedural framework for extracting kerogen from oil shale samples was referenced from the research findings of Liu et al. [50]. The three crucial steps in kerogen extraction involve acid treatment, pyrite removal, and heavy liquid separation. According to Durand [51] and Fertl [52], hydrochloric acid and hydrofluoric acid are capable of dissolving the primary mineral components in oil shale, such as quartz, feldspar, and mica, while having minimal impact on the chemical structure of kerogen. By incorporating zinc particles, pyrite can be reduced to hydrogen sulfide gas, effectively removing it from the samples. This step not only eliminates pyrite but also significantly reduces the sulfur content in the samples. Utilizing ZnCl_2_ with a density of 2.1 g/cm^3^ as the heavy liquid medium achieves efficient separation of kerogen from heavy metal ions. The extracted kerogen samples retain the same numbering as their corresponding oil shale samples.

### 4.4. Basic Properties of Kerogen

The element analysis of extracted kerogen samples was carried out by a Vario-MICRO element analyzer (producted by Elementar company, Hanau, Germany) combined with GB/T 19143-2003 [53]. An Organic Halogens Analyzer was used for the analysis of halogen elements in kerogen, with reference to GB/T 19143-2003 [53]. The CRAIC CoalPro III vitrinite reflectance measuring instrument completed the determination of the deterioration degree (vitrinite reflectance, R_O_%) of kerogen samples through GB/T 16773-2008 [54]. The kerogen content extraction experiment was carried out according to GB/T 19144-2010 [55].

Measurement uncertainty refers to the sum of random and systematic errors contained in the measurement results that cannot be accurately corrected or eliminated. It reflects the credibility or precision of the measurement result. In various analytical tests, due to multiple sources of error (such as the accuracy and stability of measurement equipment, the accuracy of measurement methods, changes in environmental conditions, and human factors), the analysis results exhibit a certain degree of uncertainty. By conducting uncertainty analysis, the impact of these errors on the analysis results can be quantified, thereby enhancing the reliability of the results. To increase the reliability of the various test and analysis results for kerogen samples, each sample underwent four repeated tests, and the uncertainty value for each analytical indicator was calculated. The specific calculation process is as follows:

(1) Basic calculation formula

(1) $$S^{2}=\frac{\sum_{i=1}^{n}{\left(x_{i}\right.-\left.X\right)}^{2}}{n-1}$$

(2) Uncertainty analysis of category A

(2) $$U_{A}=\frac{S}{\sqrt{n}}$$

The basic property parameters of the 10 kerogen samples, calculated based on Equations (1) and (2), are listed in Table 1.

### 4.5. Preprocessing of Spectral Analysis Data

The spectral data (FTIR and Raman) were first smoothed to reduce noise interference and enhance the signal-to-noise ratio. Subsequently, baseline correction was conducted using the constant baseline method, where the minimum Y value in the spectral data was selected as the baseline and automatically subtracted after fitting to ensure the accuracy of the spectral data. After baseline correction, peak picking was manually performed, with appropriate peaks selected based on the characteristics of the spectral data. The selected peaks were then integrated to calculate their peak area, full width at half maximum (FWHM), and peak center.

### 4.6. Infrared Spectrum Test and Analysis

#### 4.6.1. Infrared Spectrum Test

The experimental instrument utilized herein is a Micro-FTIR Spectrometer (model: Nicolet Continuμm™). Prior to the experiment, kerogen extracted from ten oil shale samples was thoroughly blended with potassium bromide at a ratio of 1:100. Subsequently, the kerogen was scanned for 30 s using the Micro-FTIR Spectrometer (Thermo Fisher Scientific Inc., Waltham, MA, USA) in terms of [29], with the collected wavelength range controlled between 500 and 4000 cm^−1^.

#### 4.6.2. Infrared Spectrum Analysis

In the Origin 2018 software, each infrared spectroscopy data set was processed and analyzed. For each spectrum, a normalization treatment of absorption was conducted three times to ensure data consistency and comparability. Subsequently, curve fitting operations were performed to further refine the spectral information. During the preliminary analysis, the second-derivative method of the spectrum was employed to identify the specific positions and number of spectral bands. Next, within the designated spectral regions, the number of peaks was counted. Lorentzian combinations were used to parametrically describe the shape, height, and width of the spectral bands. Finally, these spectral characteristics were compared with the characteristic curves of FTIR spectroscopy, enabling the accurate identification of spectral features corresponding to functional group types. Detailed information on the types of functional groups is provided in Table 5.

#### 4.6.3. Infrared Spectrum Fitting

To determine precise positions, bandwidths, and relative intensities, the obtained spectrum should undergo deconvolution. Common methods for FTIR deconvolution analysis include Gaussian function fitting [59], Lorentzian function fitting [60], and so forth. By applying the method proposed by He et al. [34] to perform deconvolution analysis on the original infrared spectrum of the kerogen samples, eight significant infrared spectral parameters were obtained, which can be used to reveal the molecular functional group structure of the kerogen.

(1) Aromatic structural parameters

The apparent aromaticity (*f_a_*) of coal samples is a key parameter for assessing the thermal maturity of oil shale. According to references [61,62], considering that the carbon atoms in coal samples only consist of two types: aromatic carbon (*C*_ar_) and aliphatic carbon (*C*_al_), we can calculate the fa value using the following *f_a_*:

(3) $$\frac{H_{al}}{H}=\frac{H_{al}}{H_{al}+H_{ar}}=\frac{A_{3000-2800}}{A_{3000-2800}+A_{900-700}}$$

(4) $$\frac{C_{al}}{C}=(\frac{H_{al}}{H}\times \frac{H}{C})/\frac{H_{al}}{C_{al}}$$

(5) $$f_{a}=1-\frac{C_{al}}{C}$$

where *H*_al_/H represents the ratio between the concentration of aliphatic hydrogen (*H*_al_) and the total hydrogen atom (H) concentration. This ratio is determined based on the integrated absorbance band area of aliphatic hydrogen (A_3000–2800_) within the wavelength range of 3000–2800 cm^−1^, and the integrated absorbance band area of aromatic hydrogen (*H*_ar_) (A_900–700_) within the wavelength range of 900–700 cm^−1^. Additionally, *C*_al_/C denotes the ratio between aliphatic carbon and total organic carbon; H/C represents the ratio of hydrogen atoms to carbon atoms, which is obtained through elemental analysis. As for *H*_al_/*C*_al_, it refers to the ratio of the content of hydrogen atoms in aliphatic carbon to the total content of hydrogen atoms in aliphatic carbon. Studies have shown that this ratio is approximately 1.85 in coal [63,64].

There exists a certain correlation between the thermal maturity of oil shale and its aromatic hydrocarbon content [65]. The parameter *H*_ar_/*H*_al_ serves as another indicator for measuring aromaticity and coal rank. It can be used to compare the relative abundance of aromatic functional groups versus aliphatic functional groups. Formula (6) is employed to estimate the *H*_ar_/*H*_al_ value.

(6) $$\frac{A_{ar}}{A_{al}}=\frac{A_{900-700}}{A_{3000-2800}}$$

where A_3000–2800_ is the integrated area between 3000 and 2800 cm^−1^, and it is used to estimate the total aliphatic CH content (CH_3_, CH_2_ and CH).

The internal aromatic structures of oil shale are diverse, and they vary greatly as the oil shale changes. However, structural parameters can be used to characterize the degree of development of aromatic carbon chains [66]. As the alteration of oil shale intensifies, the integration of aromatic rings also increases, while the side chains in the condensed rings become shorter, and the functional groups decrease. The specific calculation formula for the structural parameters is given in Equation (7).

(7) $${\left(R/C\right)}_{u}=1-\frac{f_{a}}{2}-\frac{\left(H/C\right)}{2}$$

(2) Aliphatic structural parameters

The length of aliphatic hydrocarbons and their branches is one of the important parameters characterizing the structural properties of low-rank oil shales. The *CH_2_*/*CH_3_* ratio can be used to determine the length of aromatic chains. An increase in this ratio implies an increase in the methylene content on the aromatic rings, accompanied by a corresponding increase in the number of aliphatic chains. Conversely, a decrease in this ratio indicates a relatively compact structure with limited space between aromatic clusters [63]. The estimation formula for the *CH_2_*/*CH_3_* intensity ratio is as follows:

(8) $$\frac{{CH}_{2}}{{CH}_{3}}=\frac{A_{2923}}{A_{2954}}$$

where the wavenumbers for *CH_3_* and *CH_2_* are located at 2954 and 2923 cm^−1^.

### 4.7. Raman Spectroscopy Testing and Analysis

#### 4.7.1. Raman Spectroscopy Testing

The kerogen sample was crushed and sieved to make its particle size reach 200 meshes, and then dried. Before the experiment, demineralization was needed. Then, its molecular parameters were accurately measured by a JY/Horiba HR 800 Raman spectrometer. Raman spectroscopy of oil shale is a crucial analytical technique, which can reveal in-depth information about its internal structure and chemical composition. This technology mainly covers two regions [67]: the first region (800–2000 cm^−1^) is closely related to the vibration modes of the main minerals and chemical bonds in oil shale, while the second region (2000–3600 cm^−1^) is usually due to the higher-order diffraction or scattering effect of the characteristic peaks in the first region. In previous studies, the secondary region was often called the “fingerprint region” because it contained rich structural details [68]. However, limited by the resolution and signal strength of the instrument, it is quite challenging to analyze these details. Therefore, this study focuses on the first-order region (800–2000 cm^−1^), which carries more information directly related to the basic structure and chemical composition of oil shale.

#### 4.7.2. Raman Spectroscopy Analysis

In the first-order region, we focus on four specific spectral bands: the G band, D_1_ band, D_2_ band, and D_4_ band (Table 6). These bands each represent crucial vibration modes and chemical bonds in oil shale, playing a vital role in identifying specific minerals, assessing the maturity of oil shale, and gaining deeper insights into depositional environments. Specifically, the G band located at 1590 cm^−1^ is closely related to the E_2g_ vibration mode of graphite, reflecting the orderliness and degree of graphitization of carbon atoms in oil shale. A higher degree of graphitization typically corresponds to a stronger G band [69], indicating a more ordered arrangement of carbon atoms in oil shale, tending towards a graphitic structure. In contrast, highly disordered carbon materials (such as oil shale) exhibit additional bands in the first-order region at approximately 1208 cm^−1^ (D_4_ band), 1350 cm^−1^ (D_1_ band), and 1560 cm^−1^ (D_2_ band) due to defects present in their crystallite lattices [70]. When assessing the Raman spectral characteristics of oil shale samples of different maturities, the maturity of the samples can be indirectly judged by analyzing the intensity ratio of the D_1_ band to the G band (A_D1_/A_G_). Generally, as the maturity of oil shale increases, the intensity of the G band enhances while the intensity of the D_1_ band relatively diminishes, resulting in a decrease in the A_D1_/A_G_ value. This trend is attributed to the gradual ordering of carbon atoms during the evolution of oil shale, forming a graphitized structure that enhances the intensity of the G band. Simultaneously, the reduction in disordered structures and defects leads to a relative decrease in the intensity of the D_1_ band.

#### 4.7.3. Raman Spectroscopy Fitting

To more accurately extract Raman spectral information of the samples, Origin 2018 software was utilized to perform Gaussian function fitting on the Raman spectral peaks within the range of 800 cm^−1^ to 2000 cm^−1^, yielding corresponding fitting results. These results encompass key parameters such as peak position difference (θ), FWHM, intensity (I), and area (A). Special attention was given to the peak position difference between the G peak and the D peak, as well as the area ratio of the D_1_ peak to the G peak [75]. The peak position difference parameter can reveal changes in the degree of graphitization within the samples. Meanwhile, the area ratio of the D_1_ peak to the G peak is widely recognized as an indicator for measuring the disorder or defect density of carbon materials. By comparing the peak position differences and area ratios among different samples, differences in their graphitization degree, disorder degree, or defect density can be assessed, further allowing inference into the thermal evolution degree of the samples.

(9) $$\theta =G-D_{1}$$

(10) $$I=\frac{A_{D1}}{A_{G}}$$

## 5. Conclusions

The text calculates key parameters such as the aromaticity, aliphatic-to-aromatic functional group ratio, and others of kerogen samples through a series of formulas. These parameters not only reveal the chemical structural characteristics of the kerogen but also provide an important basis for assessing its thermal maturity and hydrocarbon generation potential.

The H/C ratio is a crucial parameter for evaluating thermal maturity. As H/C increases, the graphitized structure decreases or becomes less ordered. When the H/C ratio in kerogen falls into the category of hydrogen-rich kerogen, the kerogen contains more aliphatic structural units (such as alkanes and cycloalkanes) or aromatic compounds rich in hydrogen substituents, resulting in a corresponding decrease in the thermal maturity of the kerogen.

The thermal maturity of kerogen is closely related to vitrinite reflectance (R_0_), aromatic structural parameters, aliphatic structural parameters, and the intensity ratio of the G peak to the D_1_ peak in Raman spectroscopy. With increasing thermal maturity, the degree of aromatization of the kerogen increases, the length of aliphatic chains changes, and the degree of graphitization enhances.

## Figures and Tables

**Table 1:** Basic property data of kerogen in oil shale samples.

Sample Number	Element (wt%)										HF (wt%)	U*_A_*__HF_	H/C	U*_A_*__H/C_	O/C	U*_A_*__O/C_	Ro (%)	$U_{A}\_R_{O}$	Kerogen (g)	U*_A_*_Kerogen
	C_daf_	U*_A_*__C_	H_daf_	U*_A_*__H_	O_daf_	U*_A_*__O_	N_daf_	U*_A_*__N_	S_daf_	U*_A_*__S_										
M-1	62.15	0.44	3.91	0.22	7.46	0.33	4.03	0.02	2.95	0.04	19.50	0.35	0.75	0.42	0.09	0.01	1.15	0.01	12	0.33
M-2	43.22	0.54	3.95	0.04	4.95	0.04	4.05	0.10	11.83	0.12	32.00	0.88	1.10	0.24	0.09	0.01	0.99	0.01	17	0.29
M-3	68.88	1.47	4.19	0.13	8.24	0.17	4.23	0.16	5.92	0.06	8.54	0.22	0.73	0.56	0.09	0.01	1.06	0.02	14	0.27
M-4	66.67	1.08	4.15	0.11	7.89	0.28	4.30	0.07	7.00	0.41	9.99	0.41	0.75	0.36	0.09	0.01	1.09	0.01	16	0.21
M-5	59.97	0.64	3.62	0.44	8.04	0.03	4.54	0.07	10.40	0.28	13.43	0.30	0.73	0.48	0.10	0.01	1.08	0.01	32	0.48
M-6	64.13	0.80	4.58	0.09	9.33	0.23	4.58	0.17	7.43	0.05	9.95	0.32	0.86	0.23	0.11	0.02	1.05	0.02	18	0.35
M-7	67.16	0.38	4.48	0.10	9.85	0.21	4.54	0.11	3.92	0.71	10.05	0.74	0.80	0.18	0.11	0.01	1.14	0.02	15	0.18
M-8	25.82	0.34	2.19	0.13	10.24	0.17	3.50	0.09	13.08	0.16	45.17	0.92	1.02	0.84	0.30	0.02	1.12	0.03	11	0.16
M-9	61.54	0.55	3.81	0.08	7.44	0.31	4.11	0.12	8.38	0.27	14.72	0.25	0.74	0.32	0.09	0.01	1.07	0.02	22	0.41
M-10	40.41	0.21	2.96	0.20	6.75	0.14	3.75	0.26	15.44	0.31	30.69	0.92	0.88	0.40	0.13	0.03	1.13	0.04	18	0.36

Note: daf: dry ash-free base; HF: halogen element. The values of uncertainty category A are all averages from four repeated experiments.

**Table 2:** FTIR analysis structural parameters.

Sample	*H*_al_/*H*	*f*_a_(FTIR)	(*R*/*C*)_u_	CH_2_/CH_3_	A_ar_/A_al_
M-1	0.80	0.68	0.29	1.62	0.26
M-2	0.55	0.67	0.11	2.24	0.67
M-3	0.74	0.71	0.28	1.75	0.43
M-4	0.72	0.71	0.27	1.52	0.38
M-5	0.68	0.73	0.27	1.78	0.52
M-6	0.76	0.65	0.25	1.97	0.30
M-7	0.70	0.70	0.25	2.17	0.45
M-8	0.50	0.72	0.13	2.03	0.72
M-9	0.69	0.72	0.27	1.81	0.50
M-10	0.70	0.67	0.23	2.41	0.41

**Table 3:** Raman spectroscopy fitting data for four representative kerogen samples.

No.	Peak	Position (cm^−1^)	Area	FWHM (cm^−1^)	*θ* (cm^−1^)	A_D1_/A_G_	A_D2_/A_G_	A_D4_/A_G_
M-3	G	1562.78	5349.68	72.38	264.01	0.64		1.43
	D_1_	1298.77	3431.70	120.29				
	D_4_	1210.00	7636.24	412.14				
M-5	G	1565.95	5863.45	74.10	260.39	0.58	0.47	
	D_1_	1305.56	3404.93	117.75				
	D_2_	1506.05	2777.89	112.30				
M-6	G	1592.36	12141.34	135.89	264.74	0.68		0.26
	D_1_	1327.62	8199.13	157.18				
	D_4_	1186.33	3200.46	302.53				
M-9	G	1567.72	6074.16	76.95	264.15	0.63	0.66	
	D_1_	1303.57	3868.11	162.29				
	D_2_	1490.62	4040.62	233.54				

**Table 4:** Deterioration temperature of samples.

Sample Number	R_O_	*T*_1_ (°C)	*T*_2_ (°C)
M-1	1.15	147	170
M-2	0.99	135	151
M-3	1.06	140	160
M-4	1.09	142	163
M-5	1.08	142	162
M-6	1.05	139	158
M-7	1.14	146	169
M-8	1.12	145	167
M-9	1.07	141	161
M-10	1.13	145	168

**Table 5:** Band distribution of different functional groups in infrared spectrum.

No.	Band Region (cm^−1^)	Functional Group	References
1	3450~3300	Hydrogen-bonded OH	[56,57]
2	3050~3030	CH_x_ stretching vibration of aromatic ring	[57,58]
3	3100~3000	Aromatic CH_x_ stretching	[56]
4	2975~2955	Asymmetric telescopic vibration of aliphatic CH_3_	[29,56]
5	2925~2919	Asymmetric telescopic vibration of aliphatic CH_2_	[29,56]
7	1800~1650	Oxygenated groups	[58]
8	1650~1410	Aromatic C=C ring stretching	[57]
9	1300~1000	Expansion deformation of phenolic resin (C-O-C)	[29,57]
10	900~700	Out-of-plane bending vibration of aromatic bond (C-H)_ar_	[56,57]

**Table 6:** First-order Raman bands of the studied coals and their vibration modes.

Band	Raman Shift (cm^−1^)	Vibration Mode
G	~1590 cm^−1^	A stretching vibration mode with E_2g_ symmetry in the aromatic layers of the graphite crystalline [71]
D_1_	~1350 cm^−1^	A graphitic lattice vibration mode with A_1g_ symmetry in-plane imperfections [71,72]
D_2_	~1560 cm^−1^	A lattice vibration analogous to E_2g_ symmetry of the G band but involving graphene layers [73,74]
D_4_	~1208 cm^−1^	sp^3^-sp^2^ mixed sites at the periphery of crystallites or to C-C and C=C stretching vibrations of polyene-like structures [73,74]

## Data Availability

Data are contained within the article.
